# Donor-Derived Cell-Free DNA in Kidney Transplantation as a Potential Rejection Biomarker: A Systematic Literature Review

**DOI:** 10.3390/jcm10020193

**Published:** 2021-01-07

**Authors:** Adrian Martuszewski, Patrycja Paluszkiewicz, Magdalena Król, Mirosław Banasik, Marta Kepinska

**Affiliations:** 1 Department of Nephrology and Transplantation Medicine, Wroclaw Medical University, Borowska 213, 50-556 Wroclaw, Poland; adert123@interia.pl (A.M.); patrycja.paluszkiewicz@op.pl (P.P.); m.banasik@interia.pl (M.B.); 2 Students Scientific Association, Department of Biomedical and Environmental Analysis, Faculty of Pharmacy, Wroclaw Medical University, 50-556 Wroclaw, Poland; m.krol1996@gmail.com; 3 Department of Biomedical and Environmental Analyses, Faculty of Pharmacy, Wroclaw Medical University, Borowska 211, 50-556 Wroclaw, Poland

**Keywords:** cfDNA, dd-cfDNA, graft failure, kidney, KTx, liquid biopsy, rejection biomarker

## Abstract

Kidney transplantation (KTx) is the best treatment method for end-stage kidney disease. KTx improves the patient’s quality of life and prolongs their survival time; however, not all patients benefit fully from the transplantation procedure. For some patients, a problem is the premature loss of graft function due to immunological or non-immunological factors. Circulating cell-free DNA (cfDNA) is degraded deoxyribonucleic acid fragments that are released into the blood and other body fluids. Donor-derived cell-free DNA (dd-cfDNA) is cfDNA that is exogenous to the patient and comes from a transplanted organ. As opposed to an invasive biopsy, dd-cfDNA can be detected by a non-invasive analysis of a sample. The increase in dd-cfDNA concentration occurs even before the creatinine level starts rising, which may enable early diagnosis of transplant injury and adequate treatment to avoid premature graft loss. In this paper, we summarise the latest promising results related to cfDNA in transplant patients.

## 1. Introduction

Most medical centres obtain great results in kidney transplantation (KTx), and graft survival within one year after transplantation is today more than 95%. A problem is the poor results of long-term follow-up after kidney transplantation, primarily due to chronic or acute antibody-mediated rejection (ABMR) [1].

The indication for KTx is end-stage kidney disease, which diabetes, high blood pressure, glomerulonephritis, polycystic kidney disease, and other diseases can lead to [2,3,4,5].

After KTx, the patient may have manifestations suggesting the need for a biopsy to finally diagnose the type of transplant injury. The biopsy evaluation is based on the current Banff classification of kidney transplant pathology, an international classification for histopathological kidney biopsy assessment. The assessment includes testing for the absence (or presence) of donor-specific antibodies (DSAs) [6,7,8].

There are some non-invasive methods of diagnosing and monitoring patients with acute rejection (AR), such as human leukocyte antigen (HLA) and non-HLA antibodies; blood gene expression profiles (Trugraf, the kidney solid organ response test—kSORT); mRNA (e.g., perforin, granzyme B, interferon (IFN)-inducible protein-10); urinary levels of chemokine (CXCL9 and CXCL10); proteomic and peptidomic signatures of AR in urine and blood samples; IFN-gamma enzyme-linked immunospot (ELISPOT) assay; or metabolomic changes [9,10,11,12,13]. Apart from these methods, monitoring of donor-derived cell-free DNA (dd-cfDNA) levels in the serum of the recipient may contribute to improved clinical outcomes after kidney transplantation. The dd-cfDNA in the blood of the recipient comes from damaged cells in the transplanted kidney. Due to rejection, the number of damaged cells, and thus the amount of dd-cfDNA, increases. The great advantage of dd-cfDNA is the possibility of non-invasive monitoring of the patient’s condition, which is currently a target in clinical trials. More clinical trials are needed for dd-cfDNA monitoring to become a standard of clinical practice [1]. There is no official Food and Drug Administration (FDA)-approved method of identifying the quantity of cell-free DNA (cfDNA). cfDNA studies have been performed in organs such as kidney, liver, heart and lung [5]. We believe that our review will inspire researchers to conduct further research. We aimed to summarise the latest research related to cell-free DNA in transplants, and particularly kidneys.

## 2. Materials and Methods

A systematic literature review was conducted per the Preferred Reporting Items for Systematic Reviews and Meta-Analyses (PRISMA) statement. Editorial comments, letters to the editor, and International Congress indexed abstracts were not considered. The comprehensive literature review was performed by a systematic analysis of literature published until October 2020. A keyword search of the PubMed database (https://www.ncbi.nlm.nih.gov/pubmed/) was conducted to find various terms: “cfDNA”, “renal transplantation”, “cell-free DNA”, and “serum cfDNA”. The publication review included living and deceased donors, as well as cellular, animal, and human studies. Prospective and retrospective clinical studies, including centre studies, meta-analyses, and review articles, were considered as well. A search of the ClinicalTrials.gov database (https://clinicaltrials.gov/) was conducted in September 2020 using the keyword “cfDNA”. Formal institutional review board (IRB) approval was not required for this study.

## 3. Characteristics of Cell-Free DNA

Circulating free DNA consists of degraded deoxyribonucleic acid (DNA) fragments that are released into the blood and other body fluids. It is present in both healthy and ill people, but in much higher concentrations in the latter [14]. The presence of cfDNA molecules in the bloodstream was first discovered in 1948 thanks to the work of two French scientists, Mandela and Metais, who studied a patient with systemic lupus erythematosus [15]. At first, this phenomenon was not considered significant or useful by clinicians. The situation changed when researchers observed differences in cfDNA concentration in healthy and ill people, which confirmed the clinical significance of the molecule. The first reports of increased cfDNA concentration concerned autoimmune diseases and leukaemia [16,17]. In subsequent scientific papers, it was noted that elevated cfDNA levels often occur in people with cancer [18]. Further research confirmed not only that tumour cells are able to release cfDNA fragments into the bloodstream, but also that the fragments have unique genetic and epigenetic changes characteristic of the tumours they originated from [19].

cfDNA exists as double-stranded, non-cell-associated DNA fragments residing in human blood and can also be detected in urine. Most cfDNA fragments are approximately 150–180 base pairs (bp) in length, which would indicate that the source of these fragments is cell apoptosis [20,21]. Larger DNA fragments, reaching up to 21 kilobase pairs (kbp) in length, also occur in patients and are most likely formed as a result of necrosis [22]. cfDNA fragments can bind to phospholipids and various proteins on the outer surface of cells. This mechanism may be associated with the absorption or release of cfDNA. Damage to DNA-binding proteins on the cell surface caused by hydrolysing enzymes can cause DNA fragments to separate from cells and release into the bloodstream [23].

### 3.1. Types of cfDNA

Based on the available literature, several types of cfDNA can be useful as biomarkers of different disease states. The most important include circulating mitochondrial DNA, also called circulating cell-free mitochondrial DNA (ccf mtDNA), circulating tumour DNA (ctDNA) [24], cell-free foetal DNA (cffDNA), and donor-derived cell-free DNA. These types are summarised in Table 1.

Circulating mitochondrial DNA consists of fragments of mitochondrial DNA (mtDNA) that are released outside the cell and persist in extracellular fluids in the form of circulating cell-free fragments. They correspond to double-stranded DNA molecules that are divided into short (less than 1 kbp) and long (up to 21 kbp) segments. Due to the very high structural similarity, with their evolutionary origins reaching back to bacterial DNA, mtDNA fragments are capable of activating the innate immunity of the body and generating inflammation through a common molecular pathway, acting in a wide variety of etiological disorders. The role of ccf mtDNA was analysed in various clinical conditions such as cancer, injury, infection, stroke, and cardiovascular disease, where it was tested as a diagnostic and predictive marker [25,26]. Recently, scholars have started to analyse mtDNA in neurological disorders as well [27,28]. Due to the higher resistance of mtDNA to nuclease-dependent degradation compared to nuclear DNA, mtDNA is maintained as ccf mtDNA in extracellular fluids. As a result of this, ccf mtDNA can serve as a potential biomarker for cell death and non-specific tissue damage, which would enable the development of screening and facilitate the prognosis of many disorders at an early stage [29].

In turn, ctDNA is fragmented DNA from a tumour that has been released into the bloodstream but is not related to cells. Since ctDNA can reflect the entire tumour genome, it has drawn attention due to its potential clinical utility. It can come directly from the tumour or from circulating tumour cells (CTCs), which are live, intact tumour cells that are released from primary tumours and reside in the blood or lymphatic system [30]. The exact mechanism of ctDNA release is not yet fully understood. Based on the available research, it can be seen that biological processes involved in the release of ctDNA include apoptosis and cell necrosis or active release from live tumour cells [31].

Another of the described types, cffDNA, is foetal DNA that freely circulates in the mother’s blood. cffDNA fragments come from placental trophoblasts. Foetal DNA is fragmented and enters the mother’s bloodstream along with placental microparticles [32]. Cell-free foetal DNA appears in a woman’s blood around the fifth to seventh week of pregnancy, and its concentration increases as the pregnancy progresses; however, it decreases rapidly after childbirth and cffDNA is undetectable in the mother’s blood just two hours afterwards. Fragments of this type of cfDNA usually have a length of about 200 bp, which allows them to be easily distinguished from much larger fragments of the mother’s DNA [33]. cffDNA analysis enables prenatal diagnostics but does not raise the risk of miscarriage due to the non-invasive way of obtaining the material (taking blood from the mother) [34].

**Figure 1:**
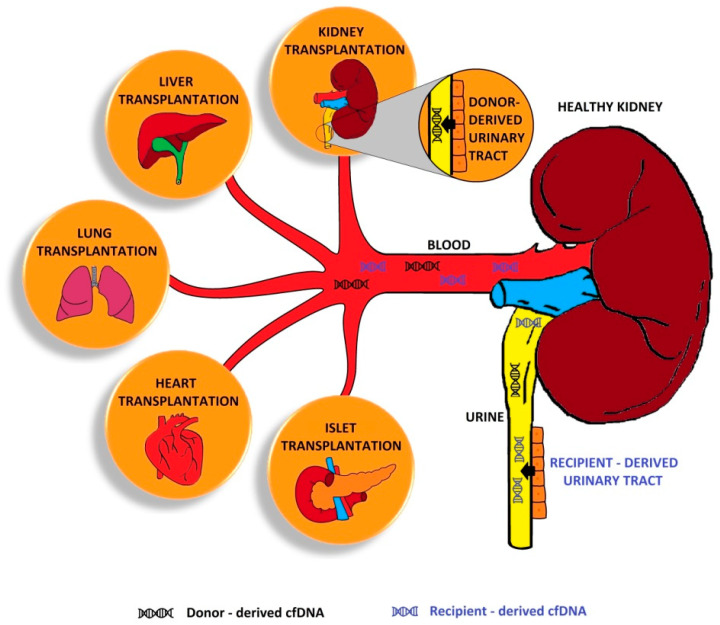
Distribution of cell-free DNA (cfDNA). Kidney transplantation (KTx) is captured here to highlight its dual function—as a kidney after other transplants and as a transplanted kidney. dd-cell-free DNA in a kidney transplant is secreted by donor-derived urinary tract and secreted into the blood.

cfDNA that is foreign to the patient and comes from a transplanted organ is defined as donor-derived cell-free DNA and can be detected both in blood and urine (Figure 1).

Initially, dd-cfDNA values increase after transplantation (>5%), but these values depend on the transplanted organ and usually decrease (<0.5%) within a week. The presence of dd-cfDNA in the patient’s blood makes it possible to assess potential complications. If the host organism rejects the transplanted organ, dd-cfDNA concentration increases more than five times compared to its level in patients without complications [35]. This increase can be detected even before other clinical or biochemical symptoms of complications. Some studies have also focused on the excretion of dd-cfDNA via urine. This is particularly vital in the case of allogeneic kidney transplants [36].

### 3.2. cfDNA in the Human Body: Origin and Removal

The concentration of cfDNA in the blood can vary significantly depending on the condition of the person being tested, and can also fluctuate as a result of disease progression. In patients diagnosed with cancer, it is usually at a level of 0–5 to >1000 ng/mL, while in healthy people it typically does not exceed 100 ng/mL. The cfDNA concentration can also vary depending on factors such as cancer type and stage. It has been proven that people in the early stages of the disease have lower cfDNA levels compared to those in advanced stages with metastases present [23]. This seems to support the idea that the amount of cfDNA can illustrate the tumour interaction microenvironment or describe cancer biology and metabolism. On the other hand, the increasing concentration of these molecules may be due to the increasing number of cells undergoing apoptosis or related to the burden on the body of developing cancer.

cfDNA formation and release characteristics still require further research to investigate the complex nature of these processes, along with the removal of these molecules from the body. The research carried out so far indicates that the half-life of cfDNA in the bloodstream is estimated to be between 16 min and 2.5 h [37,38]. Unfortunately, this also requires further confirmation and must be checked under various environmental conditions and in patients in different states of health. It is known that cfDNA can enter the liver or spleen and be degraded by macrophages located inside them [39] as a result of the enzyme activity of DNase I [40], or can be removed from the body through filtration in the kidneys and excreted via urine [41]. As already mentioned, cfDNA can bind to proteins on the surface of cells and be transported to others for possible degradation or transport to the cell nucleus. The binding of cfDNA to receptors on the surface of cells depends on factors such as pH and temperature, and can be inhibited by various substances [42]. Therefore, the speed of its removal from the human body may depend on the speed of its binding and cellular uptake.

## 4. cfDNA Detection

Although serum and plasma remain the most popular materials for cfDNA analysis, there have been cases of clinical use of other biological materials; this is because cfDNA derived from fluids that have increased contact with the tumour microenvironment can be more indicative of the disease status. As mentioned before when discussing cfDNA characteristics, its molecules can be removed via urine, so urine can be used for testing as well [43]. Other means of excretion include saliva, sputum, faeces, and cerebrospinal fluid [44,45,46,47]. According to the latest knowledge, serum samples have higher concentrations of cfDNA than plasma. Serum testing can also be used to better detect specific mutations in molecules that come from tumours. Research also suggests that cfDNA present in serum is not subject to such severe destructive processes as in plasma [48].

### 4.1. Pre-Analytical Steps

**Figure 2:**
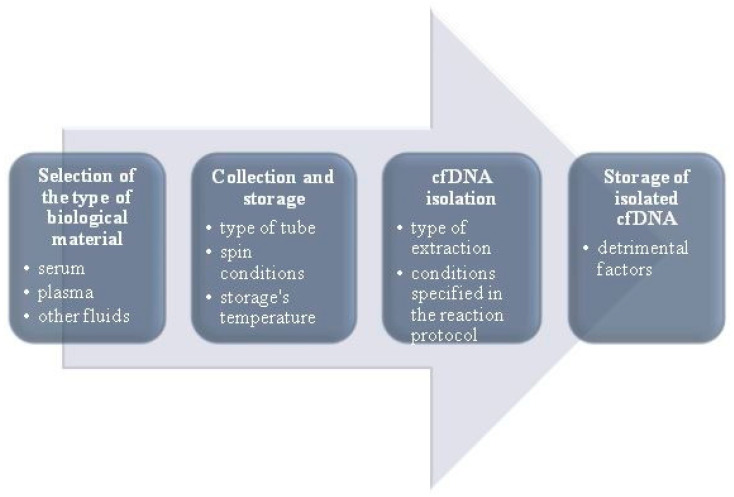
Pre-analytical stages to optimise the extraction process (modified based on Bronkhorst et al. [37]).

Assessment of cfDNA concentration and/or other biomarkers in plasma or serum is also called “liquid biopsy”. Recently, it has become one of the most important clinical analyses aimed at early detection of cancer, as well as monitoring its course, predicting relapses, assessing cancer therapeutic resistance, and conducting prenatal tests. Comprehensive liquid biopsy analysis can additionally be a tool for assessing tumour burden, as well as a cancer’s molecular features [49]. This is why carrying out cfDNA analysis procedures properly is so vital. Unfortunately, there are some challenges and difficulties in extraction, and the process itself is not easy due to cfDNA fragmentation. Depending on the extraction method, obtaining cfDNA fragments of different length from the same sample can be another difficulty. Apart from obtaining different-sized cfDNA fragments, different extraction methods can also result in various concentrations of the molecules tested [50,51]. Since the concentration of cfDNA molecules is often very low, any loss of biological material from the sample reduces the sensitivity of further molecular analyses. As such, choosing the right extraction method to ensure the highest efficiency is extremely important. Additionally, cfDNA extraction efficiency can be increased by using optimal pre-analytical steps [52]. Figure 2 shows pre-analytical stages which can help optimise the extraction process [37].

Extraction is followed by the next stage of cfDNA analysis, detection. Real-time polymerase chain reaction (RT-PCR) is a commonly accepted detection method [53]. It detects all types of free and protein-bound cfDNA. An alternative method of direct immunological detection of nucleosomal DNA in serum and plasma has also been described. This method only captures the histone-related fraction, which is the main part of circulating DNA. A ready-made enzyme-linked immunosorbent assay (ELISA) kit (e.g., Cell Death Detection ELISAplus) can be used for this purpose, utilising anti-H1, H2A, H2B, H3, and H4 histone antibodies and anti-DNA antibodies that bind to single- and double-stranded DNA. Therefore, the test can detect both mono- and oligonucleosomes [54].

### 4.2. Problems with cfDNA Detection

Unfortunately, we may also encounter difficulties at the cfDNA detection stage. The sensitivity of technologies used to detect cancer cfDNA is expressed using the so-called mutant allele fraction (MAF) range, i.e., the average fraction of the mutated allele, which is a measure that determines the ratio of the number of mutated to wild-type alleles in the sample. The detection limits of MAF in traditional qPCR range from 10 to 20% [37]. Many PCR-based variants increase sensitivity, such as allele-specific quantitative polymerase chain reaction (AS qPCR) [55]. In turn, digital PCR (dPCR) methods can quantify cfDNA with exceptional sensitivity, up to 0.001–0.05% MAF [56,57]. These tests are based on differences in the binding affinity of mutant and wild-type alleles, which require primers or probes targeting specific mutations, and the multiplex ability is limited in this case [58].

There are also other methods for detecting cfDNA, such as whole-genome sequencing, including Plasma-Seq [59] and Fast Aneuploidy Screening Test Sequencing System (FAST-SeqS) [60], which require relatively large amounts of cfDNA to be detected (5–10% MAF) [37]. Fortunately, it has already been shown that the MAF detection limit can be reduced to as low as 0.1% by minimising background sequencing error levels and increasing the signal-to-noise ratio of the samples [61,62]. Sampling noise can also be reduced by implanting selective cfDNA scavengers, which simultaneously increases the efficiency [61]. Additionally, the method sensitivity can be increased by using exome sequencing, digital sequencing, and PCR enhancers (e.g., Safe-Seqs) [63].

Such technical limitations can be overcome by examining the genomic features of cfDNA that are significantly altered, including diverse methylation patterns and post-translational histone modifications [37]. The use of this data would increase the likelihood of cancer detection and help determine tumour location, and could also be helpful in identifying cancers of unknown origin. Studies should first be conducted on large groups of patients before their clinical utility can be confirmed.

Among the key elements of cfDNA detection are the sensitivity and specificity of tests due to their usefulness in screening and distinguishing between ill and healthy people. Therefore, before introducing these methods for diagnostic screening, the sensitivity of the available tests should be increased. These methods already show sufficient sensitivity in giving prognoses and assessing disease severity. In addition, they can also be vital when choosing a treatment method, assessing the response to a chosen therapy, monitoring minimal residual disease, and predicting relapses.

### 4.3. Distinguishing dd-cfDNA from Recipient cfDNA

Quantification of cfDNA in circulating blood derived from a transplanted organ is a powerful approach to monitoring post-transplant injury. Detection of dd-cfDNA is based on chimerism, i.e., the presence of cells in the transplant recipient that are genetically different from the cells of the transplant donor [64]. The potential of dd-cfDNA was first tested in liver and kidney transplant recipients. Sex-specific chromosome differences were used to distinguish donor and recipient cfDNAs. Male donor cfDNA was detected in 23 female kidney or liver transplant recipients using chromosome Y (ChrY)-specific quantitative PCR (qPCR) [65]. This method is characterised by high sensitivity, short lead time, and relatively low cost; however, it cannot be applied in all patients, as women receiving organs from male donors are only a small fraction of the total recipient population.

Gadi et al. [66] used the qPCR test targeted at specific polymorphisms in human leukocyte antigen (HLA) sequences. Compared to the detection of ChrY, this method is not gender-specific and therefore has wider application. Digital droplet PCR (ddPCR) [67] can be used to quantify dd-cfDNA as well. Similarly to qPCR, ddPCR can also be used to detect genetic differences between donors and recipients, including single nucleotide polymorphisms (SNPs), ChrY, and insertions or deletions (InDels). ddPCR displays greater sensitivity, precision, and completeness of quantification. This technique is based on a variation in the number of base pairs, making the use of InDels more sensitive [64].

Today, genome transplant dynamics (GTD) uses different SNPs to distinguish between host and donor molecules to determine whether dd-cfDNA from a transplanted organ is present in the recipient’s bloodstream. Unfortunately, this procedure requires genotyping for both recipient and donor, and clinical practice shows that information about the donor’s genotype is often difficult to obtain [68]. Various algorithms have been developed to overcome this problem [68,69]. Additionally, next-generation sequencing (NGS) assays are starting to be used to quantify dd-cfDNA, which suggests great clinical utility and further benefits [70,71].

SNPs or InDels that are present in one or two alleles of the donor, and not present in the recipient genome, may be useful for the detection of dd-cfDNA. SNPs that are highly variable in the population are particularly useful, as this increases the probability that donor DNA can be distinguished from recipient DNA, and thus dd-cfDNA from recipient cfDNA. Another option for SNP quantification is massive parallel shotgun sequencing, which is a form of NGS [72]. While this method is characterised by high sensitivity, it is also very expensive and has a long implementation time.

## 5. cfDNA in Transplantations

Elevated serum dd-cfDNA is suggested as a biomarker of rejection in liver, lung, and heart transplant (HT) recipients [73,74,75].

Krenzien et al. [76] examined the hypothesis that liver transplant recipients have high levels of cfDNA due to ischemic reperfusion injury (IRI). The population group included 50 patients, who had elevated cfDNA levels in the first week after transplantation. The authors concluded that increased cfDNA in liver transplant recipients is associated with worse 1-year survival. They also found granulocytes and eosinophils in liver biopsies of patients with high cfDNA, which was in line with the increased level of neutrophils in their blood. In another prospective observational multicentre cohort study, the level of dd-cfDNA in the plasma of 115 liver transplant patients was tested. It was determined that dd-cfDNA measurements are better than standard patient liver monitoring. In addition, dd-cfDNA may help to identify rejection 8 to 15 days before a biopsy-proven rejection with sensitivity and specificity of about 90% [77].

Sayah et al. [78] analysed dd-cfDNA in blood obtained from lung transplant patients. dd-cfDNA was elevated in patients with acute cellular rejection and a cut-off of 0.87%, with sensitivity at 73.1% and specificity at 52.9%. To detect AR in lung transplant recipients, a transbronchial lung biopsy via fibreoptic bronchoscopy is typically performed. More than 85% of lung transplants show an HLA mismatch of at least four out six alleles, while only less than 0.1% show a donor–recipient HLA match of six out six alleles [79]. Zou et al. [80] developed a test method based on phenotypic HLA differences between recipient and donor DNA. They developed a panel of DNA probes in which each probe targets a unique HLA allele sequence. After transplantation, donor- and recipient-specific probes are matched based on the differences in HLA loci, after which ddPCR quantifies the amount of dd-cfDNA in significant excess of recipient cfDNA. It was determined that the dd-cfDNA level was significantly elevated in patients with AR (*p* < 0.01). dd-cfDNA can be used as a non-invasive early biomarker of acute rejection, detected using a peripheral blood sample.

Richmond et al. [81] analysed 624 biopsy-paired blood samples obtained from seven centres. They concluded that dd-cfDNA could be useful for ruling out acute rejection after HT in paediatric and adult population groups with a cut-off value of 0.16% (sensitivity: 75%; specificity: 79%). Non-invasive monitoring of dd-cfDNA levels provides an alternative to endomyocardial biopsy as a standard for assessing rejection in HT recipients. Iwijn De Vlaminck et al. [73] analysed dd-cfDNA using 565 plasma samples obtained from HT patients. It was concluded that the increased dd-cfDNA level was in line with acute rejection, with sensitivity and specificity comparable to biopsy. Khush et al. [82] analysed blood samples from HT patients in a multicentre study with a reference population of 646 patients. The dd-cfDNA median level in this population was 0.07%. In patients with acute rejection, the median level of dd-cfDNA was 0.17%. Using a threshold of 0.2%, the authors determined that the sensitivity of this method for detecting rejection was 44%.

Despite the clinical success of pancreatic islet transplants, results may be worse due to the early death of cells after intraportal infusion. After transplantation, islets are under-oxidised, exposed to alloimmunity and instant blood-mediated inflammatory response (IBMIR), leading to the loss of a significant proportion of the transplant. Alloimmunity is controlled by measuring DSA. Pre-transplantation DSA was associated with failure of the transplant, but not all studies have proven this [83]. A limiting factor in predicting early and long-term function of a transplant is the impossibility of determining cell death after transplantation. During 2014–2016, Gala-Lopez et al. [84] conducted research involving isolation of pancreatic islets and allotransplantation. A total of 100 islet allotransplantations were performed on 83 patients. Statistical analysis was carried out on data of 37 patients who met the relevant criteria, such as receiving their first transplant or a second transplant more than a year after the previous one. The beta cell-specific level of cfDNA in plasma was measured 1 h, 24 h, 7 days, and 1 month after transplantation; the function of the transplant and the requirements for exogenous insulin were examined as well. The results showed that beta cell-specific cfDNA measured 24 h after transplantation correlated with the transplant functioning immediately after the procedure. An hour after transplantation, patients both with and without cfDNA signals had similar initial transplantation function, which was assessed using monthly exogenous insulin requirements and C-peptide levels (0.14 vs. 0.11 U/kg per day, *p* < 0.05) and 1 month stimulated C-peptide levels (0.92 vs. 1.3 nmol/L, *p* < 0.05). Patients with cfDNA detected 24 h after transplantation had a significantly higher monthly insulin demand, higher absolute insulin consumption, and lower C-peptide levels stimulated for one month. Beta cell-specific cfDNA measured 24 h after transplantation may be a predictive factor for islet transplantation.

By September 2020, five clinical trials related to cfDNA in transplantation were registered in the ClinicalTrials.gov database (Table 2). More clinical trials with study groups including kidney transplant recipients are required.

## 6. cfDNA in Kidney Transplantation

**Figure 3:**
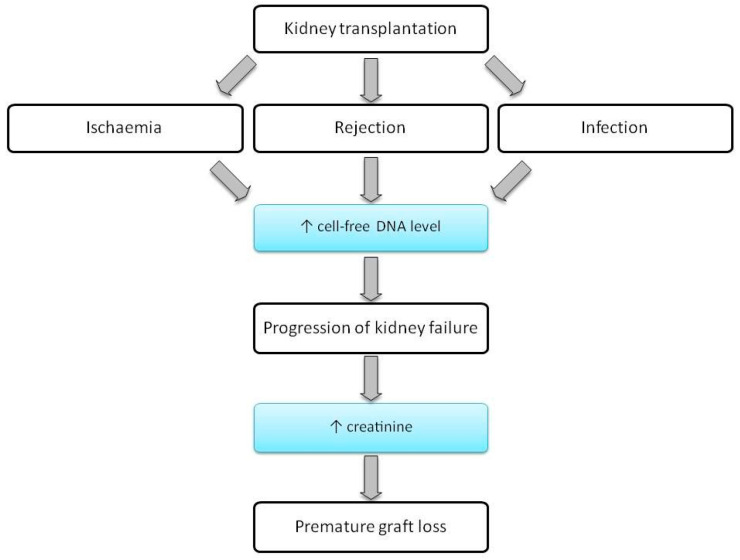
A flow chart showing the progression from KTx to premature graft loss. cfDNA may be a biomarker indicative of graft injury at a very early stage.

Currently, the only reliable method of detecting the causes of kidney injuries is an invasive test, a transplant biopsy. Developing a non-invasive diagnostic method to detect damage after organ transplantation is a clinical need that has not been met so far. It has been assessed that cfDNA may be a potential substitute for the donor’s acute organ transplantation biomarker and AR biomarker (Figure 3).

Early non-invasive detection of an increase in cfDNA level could reduce the number of unnecessary biopsies. In addition, it would allow clinicians to modify immunosuppressive therapy at the proper time. The immunosuppressive drugs used by kidney transplant recipients often include tacrolimus. It has been shown that dd-cfDNA can be used to assess the effective tacrolimus trough concentrations in the livers of kidney transplant recipients. The clinical utility of the dd-cfDNA test needs to be better defined. It is missing the distinction between immunological and non-immunological factors, which can be rectified with additional analysis of donor-specific antibodies as the main immunological factor causing graft injury [85,86,87].

Dauber et al. [5] conducted a study in which they determined the dd-cfDNA levels in serum using quantitative RT-PCR, which analysed InDels in patients after KTx. In patients with AR, the dd-cfDNA level was 5.24% of the total cfDNA in the recipient’s plasma, while in the remaining patients it was 3.74% lower. Analysing InDels seems to help differentiate AR from other causes of rejection after KTx.

Some studies have shown that T cell-mediated rejection (TCMR) is not always associated with increased dd-cfDNA in the serum [88,89]. Stites et al. [90] analysed dd-cfDNA levels in patients with T cell-mediated rejection grade IA (TCMR IA), as well as borderline conditions (inflammation that does not qualify as TCMR), after biopsy. The authors demonstrated the presence of a significantly higher amount of cfDNA in the serum of patients with reduced estimated glomerular filtration rate (eGFR) and patients with DSA present in the serum. They suggested the potential use of cfDNA assays to complement the Banff classification of kidney allograft pathology based on histologic biopsy assessment.

A study by Bloom et al. [88] on 102 patients showed a statistically significant difference in dd-cfDNA levels in patients with active rejection compared to the non-rejection group. It is assumed that the probability of acute rejection increases when the dd-cfDNA level exceeds 1%. Another study of 107 plasma samples of kidney transplant patients, looking for cfDNA derived from the donor organ (dd-cfDNA) using NGS was carried out by Gielis et al. [91]. A total of 1036 plasma samples were tested and a threshold of 0.88% was established. According to the authors, rejection had a higher diagnostic value in the case of elevated creatinine level, but an increase in dd-cfDNA was also observed. The increased dd-cfDNA was often associated with pyelonephritis and acute rejection. Despite this, the authors did not find any correlation between kidney function and dd-cfDNA percentage in a 3-month follow-up after transplantation (determining serum creatinine or eGFR at the same time). The study showed that dd-cfDNA percentage has a similar diagnostic value as serum creatinine in AR prognosis. There was also no significant increase in dd-cfDNA in the case of cytomegalovirus (CMV) or bovine kobuvirus (BKV) infection. In their previous work [92], they studied patient samples using PCR to determine the dd-cfDNA kinetics after transplantation and the threshold value. The level decreased exponentially in plasma within 10 days after transplantation and the threshold value was 0.88%. The authors also reported that recipients with longer warm ischaemia time (WIT) had higher dd-cfDNA levels in the first day after transplantation.

dd-cfDNA is used in the United States to detect rejection in kidney transplant recipients and has been reimbursed under Medicare (the US government social insurance programme) since October 2017. Huang et al. [89] analysed 63 adult KTx recipients with suspected allograft rejection. For this purpose, dd-cfDNA measurement and allograft biopsy were performed. In this analysis, 43% of the patients had DSA, and biopsy showed rejection in 54% of cases. The percentage of dd-cfDNA was found to be higher (median 1.4%) in recipients with ABMR compared to recipients without rejection (median 0.38%) and recipients with T cell-mediated rejection (median 0.27%). The authors suggested that due to unclear reasons, cfDNA tests had false positive results in patients with T cell-mediated rejection. It seems that while cfDNA may have broad application for detecting the presence of ABMR, the existing scientific data of dd-cfDNA are limited and further research is needed.

Sigdel et al. [93] carried out a study to measure dd-cfDNA levels by SNP-based massively multiplexed PCR (mmPCR) using NGS in 300 plasma samples collected from patients after KTx. In this retrospective analysis, the mmPCR-NGS method had more than 80% sensitivity and more than 70% specificity in detecting dd-cfDNA levels (cut-off >1%), higher than in the case of eGFR (cut-off <60 for AR). Median cfDNA in the AR group was 2.32% and in the non-rejection group was 0.47%, however there was no difference between TCMR and ABMR groups.

Zhang et al. [94] studied blood samples of patients a median of 3 years after kidney transplantation with ABMR and stable graft function to determine the presence of cfDNA and attempted to assess the impact of dd-cfDNA on ABMR pathological severity and prognosis. Biomarker levels were measured using a targeted next-generation sequencing assay. The mean amount of dd-cfDNA in plasma was 2.4% in the ABMR group, which was higher than in the stable allograft group (0.65%). There was no significant difference in cfDNA levels between acute and chronic ABMR; however, in patients with ABMR, cfDNA levels were elevated compared to those without rejection. The cut-off point was 1% of the cfDNA value.

Hurkmans et al. [95] published a case report in which they described a patient with increased dd-cfDNA to 23% of total cfDNA after kidney transplantation. The increased dd-cfDNA suggested transplant rejection, which was confirmed by biopsy. The patient had co-occurring melanoma, which was treated with an immune checkpoint inhibitor, which probably stimulated allograft rejection. The dd-cfDNA level probably increased because of the presence of cancer. This may suggest a lower sensitivity of the marker.

Oellerich et al. [4] indicated an inaccuracy in the fractional determination of cfDNA levels and suggested measuring this biomarker by absolute quantification of dd-cfDNA (copies per mL of plasma). They conducted a single centre study of patients after KTx. Five days after KTx, the average amount of dd-cfDNA fraction was about 0.6% regardless of the donor, and the absolute dd-cfDNA amounted to 98 cp/mL from non-living and 68 cp/mL from living donors. Values above 0.50% and 50 cp/mL are of practical importance. In patients with biopsy-proven rejection (BPR), the dd-cfDNA median was 0.57% and 82 cp/mL, while in stable patients it was 0.29% and 25 cp/mL. The authors suggested that this biomarker may detect inadequate immunosuppression because it was increased in patients with lower tacrolimus levels. Measuring dd-cfDNA (%) in comparison with copy number variation (CNV) (cp/mL) in the receiver operating characteristic (ROC) analysis shows differences (0.73 vs. 0.83, respectively) in the area under the curve (AUC). Whitlam et al. [96] concluded that the determination of dd-cfDNA fraction or absolute quantification of dd-cfDNA may be equally helpful in diagnosis. In the diagnosis of ABMR, determination of dd-cfDNA (%) and dd-cfDNA (cp/mL) has a sensitivity of 0.85 for both and a specificity of 0.75 and 0.79, respectively.

Shen et al. [97] analysed 77 blood samples of 28 AR patients (5 with ABMR and 23 with TCMR). The amounts of total cfDNA and dd-cfDNA were measured before and after anti-rejection therapy. Both parameters significantly decreased. Serum creatinine showed no significant difference in either subgroup after therapy. dd-cfDNA was measured 2 weeks after the end of therapy, and results showed no significant differences. The same comparative analysis was performed for creatinine, and a slight decrease was observed 2 weeks after the end of anti-rejection therapy.

Zhou et al. [98] examined 32 samples of peripheral blood from 30 KTx patients. The mean dd-cfDNA ratio was 1.17% (highest, 3.53%; lowest, 0.23%) and was more statistically significant in patients with rejection.

An important problem in clinical practice is the difficulty of early diagnosis of kidney injury which may have a bad influence on chronic kidney disease (CKD). Patients with CKD may consequently require KTx. Watson et al. [99] developed the Kidney Injury Test (KIT) after analysing 397 urine samples collected from CKD patients. They measured 6 biomarkers: cfDNA, methylated cfDNA (m cfDNA), CXCL10, clusterin, total protein, and creatinine. The KIT can be used for monitoring of CKD patients.

In some patients, immunoglobulin A (IgA) nephropathy recurs after KTx [100]. Yang et al. [101] studied urine samples of 34 patients with IgA nephropathy using the KIT developed by Watson et al. It turned out that the KIT panel may be useful in distinguishing healthy patients from those with IgAN; however, the cfDNA values were the best predictor.

In another multicentre prospective study [102], KIT was used to analyse urine samples collected just before kidney allograft biopsy in paediatric and adult KTx patients. They measured 6 biomarkers in 364 urine samples which were paired with kidney transplant biopsies of transplant recipients (mainly in the case of stable allograft function and acute rejection patients). These biomarkers were cfDNA, methylated cfDNA (m-cfDNA), clusterin, CXCL10, creatinine, and total protein. Based on these, the authors developed the quantitative Q score (QiSant score) with a scale of 0–100. It has been observed that the QiSant score can determine the occurrence of TCMR and ABMR. It can also predict an episode of rejection up to 200 days before biopsy-confirmed rejection. The KIT enabled the diagnosis of AR with 96% accuracy, as well as early diagnosis of AR even before increased serum creatinine was present. Nolan et al. [103] analysed 152 urine samples from patients with stable allograft function (STA) and 71 samples from patients who suffered AR after KTx, indicating the levels of 6 biomarkers using the QiSant score and blinded to clinical information. The Q score test in this study successfully distinguished patients with AR (Q score ≥ 32) and STA (Q score < 32) with sensitivity and specificity of more than 90% in adult and paediatric groups. There were no statistically significant differences between patients with ABMR and TCMR. Urinary tract infection (UTI) can occur in KTx patients and occurs in 17% of patients within 6 months after the procedure. Later, UTIs also increase the risk of death in these patients [104]. Using next-generation sequencing, Burnham et al. [105] tested 141 urine samples from KTx patients for cfDNA; this included two groups, bacterial (99 samples) and viral (42 samples). All patients with BKV infection had high levels of cfDNA.

The level of dd-cfDNA in urine correlates with the protein/creatinine ratio (r = 0.48; *p* < 0.05) and glomerular filtration index (r = 0.52; *p* < 0.05). It was most sensitive to acute allograph damage. The measurement of dd-cfDNA in urine may be a sensitive marker of acute injury in the donor organ [106].

## 7. cfDNA in Kidney Diseases

Homolová et al. [107] studied the amount of extracellular DNA (ecDNA) in plasma in animal models of acute kidney injury (AKI). The authors identified three subtypes of ecDNA: mtDNA, nuclear ecDNA (ncDNA), and total ecDNA. In AKI induced by bilateral ureteral obstruction and glycerol, higher total ecDNA was observed. Similarly, in bilateral ureteral obstruction-induced AKI, ncDNA was increased compared to sham (placebo operation without ligation, excision, or clamping). Based on this study, it was concluded that AKI pathogenesis significantly affects the increase in ecDNA in plasma and may be useful in diagnosing AKI.

It was reported that cfDNA levels are elevated in type 2 diabetes mellitus (T2DM). In addition, patients with retinopathy have higher levels of cfDNA than those without it [108]. Many T2DM patients can develop diabetic kidney disease (DKD). In a prospective study, blood samples from patients with DKD were analysed to determine the serum level of cfDNA with a 3-year follow-up. cfDNA in serum was higher in patients with DKD progression (960.49 ng/mL), and it was defined as decreased eGFR or urinary albumin-to-creatinine ratio compared to the group without disease progression (824.51 ng/mL). As such, serum cfDNA is associated with DKD and can be used as a predictor in patients with T2DM who develop DKD [109].

A study of 131 patients diagnosed with CKD was carried out by Chang et al. [110], which showed a correlation between low plasma cfDNA and cf mtDNA concentration in urine and favourable treatment results within 6 months; as such, they are prognostic factors. They are non-invasive biomarkers of CKD and make it possible to monitor its progress.

Immunohistochemical testing and biochemical characterisation of tumours are currently used as the basis for making diagnoses and determining the prognosis for patients with kidney cancer. Many researchers are intensively searching for new diagnostic and prognostic markers in easily accessible and retrievable biological samples, especially blood and urine. ctDNA is highly specific and can be used for diagnosis and as a prognostic and predictive factor [111]. In body fluids other than blood, ctDNA is not diluted; it reaches higher concentrations and its presence is a result of necrotic changes (and/or it is actively excreted). The use of body fluids which are in direct contact with cancer cells to measure ctDNA may provide an alternative to measuring ctDNA in the blood, as some cancer types only secrete small amounts of ctDNA into it. The type of biological material in which a high level of ctDNA is detected suggests the cancer location; in addition, the marker is also an indicator of genetic and epigenetic changes in the cancer tissue. As such, detecting and quantifying cancer cells becomes possible.

Lasseter et al. [112] studied the possibility of using m-cfDNA immunoprecipitation sequencing (cfMeDIP-seq) to detect renal clear cell carcinoma (RCC) after isolating cfDNA plasma. The results of the analysis showed that the use of sequencing increased sensitivity to 52% (from 39%), whereas in the case of metastatic RCC, the sensitivity was 100% with 88% specificity. cfMeDIP-seq monitoring may also be useful as a predictive marker.

Bacon et al. [113] tested blood samples from patients with metastatic RCC to determine the usefulness of cfDNA as a predictive biomarker. Patients with metastatic RCC and elevated serum cfDNA had significantly shorter progression-free survival (PFS) and overall survival (OS) with first-line therapy.

Rouvinov et al. [114] analysed blood samples collected from 23 patients to verify whether cfDNA could be a predictive biomarker in the treatment of metastatic RCC (mRCC). The cfDNA level was measured using a fluorometric assay. An elevated cfDNA level before targeted mRCC therapy can be associated with worse PFS. Skrypkina et al. [115] carried out a study in which they tried to check the usefulness of measuring cfDNA levels in plasma samples as a kidney cancer biomarker (involving 27 patients with carcinoma and 15 healthy donors). cfDNA levels were measured using two methods: SYBR Green I fluorescence and quantitative real-time PCR (qRT-PCR). For the results obtained using fluorescence, the median cfDNA level was 235.55 ng/mL in patients with renal cancer, while in the healthy group it was 53.66 ng/mL. For the qRT-PCR method, the values were also higher for renal carcinoma patients (median 80.97 ng/mL) than for healthy donors (median 35.1 ng/mL). The authors also studied the methylation of specific genes in these samples (*FHIT*, *APC*, and *RASSF1*) and concluded that cfDNA with methylation can be a useful addition to serological tumour markers in detecting renal carcinoma. cfDNA alone cannot be a specific biomarker for detecting kidney cancer [31].

In their original article, Lu et al. [116] concluded that cf mtDNA (measured using qRT-PCR) is higher in patients with mRCC compared to those with non-metastatic RCC and healthy people. They also suggested that cfDNA can be a prognostic marker in non-metastatic RCC.

## 8. Conclusions

Since 1998, there has been great interest in cfDNA plasma testing in molecular diagnostics for cancer detection and prenatal diagnosis. It was eventually discovered that dd-cfDNA can be detected in the plasma of transplant recipients. The method of genotyping the constitutional DNA of transplant donors and recipients provided genotyping information which allowed researchers to analyse the sequencing of DNA in plasma and study the polymorphisms specific to donors. Different methods have established an average value of less than 1% for cfDNA in healthy individuals, whereas during organ rejection, cfDNA levels increase to an average of 3–4% of the total cfDNA.

Assessing the level of dd-cfDNA seems to be a minimally invasive and sensitive method. Compared to biopsy, the waiting time is much shorter as well; however, false positive results can occur for KTx patients with coexisting diseases and other cancer types. The results may also be questionable if there has been post-allograft retransplantation.

The presence of the donor genome in a transplanted organ can be used to monitor the condition of the transplanted organ. Treatment leads to a decrease in cfDNA levels. If detected quickly, rejection can be treated at an early stage before complete rejection occurs. Future multicentre studies and clinical trials on detecting dd-cfDNA (in urine and serum) in KTx patients with suspected graft rejection are still required. The methods to be used in such studies are vital and any samples obtained should be in line with the biopsy results.

## Figures and Tables

**Table 1:** Types of cell-free DNA (cfDNA) and their potential applications.

Types of Cell-Free DNA	Abbreviations	Potential Application	References
Circulating cell-free mitochondrial DNA	ccf mtDNA	Diagnostic and predictive marker in various disease states, marker of cell death and non-specific tissue damage	[25,26,27,28,29]
Circulating tumour DNA	ctDNA	Marker in oncological diagnostics, monitoring of tumour development	[30,31]
Cell-free foetal DNA	cffDNA	Prenatal diagnostics, detection of foetal defects	[32,33,34]
Donor-derived cell-free DNA	dd-cfDNA	Evaluation of post-transplant complications	[35,36]

**Table 2:** The five clinical trials of cfDNA in transplantation downloaded from ClinicalTrials.gov database (https://clinicaltrials.gov/) with terminated, completed, and active, not recruiting status.

NCT Number	Title	Condition	Actual Enrolment	Recruitment Status	Location	Age of Participants ^1^
NCT03765203	Utility of a Novel Dd-cfDNA Test to Detect Injury in Renal Post-Transplant Patients (QIDNEY)	Kidney transplant failure	175 participants	Completed	United States	Child, adult, older adult
NCT04271267	Cell-free DNA as a Biomarker After Lung Transplantation	Lung transplant rejection	126 participants	Completed	No data	Adult, older adult
NCT02424227	Non Invasive Blood Test To Diagnose Acute Rejection After Kidney Transplantation (DART)	Kidney transplant recipients	401 participants	Active, not recruiting	United States	Adult, older adult
NCT01985412	Genome Transplant Dynamics: Non-invasive Sequencing-based Diagnosis of Rejection (GTD)	Cardiac transplant rejection; lung transplant rejection	65 participants	Completed	United States	Child, adult
NCT02109575	Quantitative Detection of Circulating Donor-Specific DNA in Organ Transplant Recipients (DTRT-Multi-Center Study) (DTRT)	Cardiovascular disease; acute rejection of cardiac transplant; cardiac transplant rejection; heart transplant failure and rejection	no data	Active, not recruiting	United States	Child, adult, older adult

^1^ Child: new-born to 17 years old; adult: 18–64 years old; older adult: more than 65 years old.

## Data Availability

No new data were created or analysed in this study. Data sharing is not applicable to this article.

## References

[1] Viklicky O., Novotny M., Hruba P. (2020). Future developments in kidney transplantation. Curr. Opin. Organ Transplant..

[2] Maxeiner A., Bichmann A., Oberländer N., El-Bandar N., Sugünes N., Ralla B., Biernath N., Liefeldt L., Budde K., Giessing M. (2019). Native Nephrectomy before and after Renal Transplantation in Patients with Autosomal Dominant Polycystic Kidney Disease (ADPKD). J. Clin. Med..

[3] Bellini M.I., Courtney A.E., McCaughan J.A. (2020). Living Donor Kidney Transplantation Improves Graft and Recipient Survival in Patients with Multiple Kidney Transplants. J. Clin. Med..

[4] Oellerich M., Shipkova M., Asendorf T., Walson P.D., Schauerte V., Mettenmeyer N., Kabakchiev M., Hasche G., Gröne H., Friede T. (2019). Absolute quantification of donor-derived cell-free DNA as a marker of rejection and graft injury in kidney transplantation: Results from a prospective observational study. Arab. Archaeol. Epigr..

[5] Dauber E., Kollmann D., Kozakowski N., Rasoul-Rockenschaub S., Soliman T., Berlakovich G.A., Mayr W.R. (2019). Quantitative PCR of INDELs to measure donor-derived cell-free DNA—A potential method to detect acute rejection in kidney transplantation: A pilot study. Transpl. Int..

[6] Solez K., Colvin R.B., Racusen L.C., Haas M., Sis B., Mengel M., Halloran P.F., Baldwin W., Banfi G., Collins A.B. (2008). Banff 07 Classification of Renal Allograft Pathology: Updates and Future Directions. Arab. Archaeol. Epigr..

[7] Jeong H.-Y. (2020). Diagnosis of renal transplant rejection: Banff classification and beyond. Kidney Res. Clin. Pract..

[8] Haas M., Loupy A., Lefaucheur C., Roufosse C., Glotz D., Seron D., Nankivell B.J., Halloran P.F., Colvin R.B., Akalin E. (2018). The Banff 2017 Kidney Meeting Report: Revised diagnostic criteria for chronic active T cell–mediated rejection, antibody-mediated rejection, and prospects for integrative endpoints for next-generation clinical trials. Arab. Archaeol. Epigr..

[9] Thongprayoon C., Vaitla P., Craici I.M., Leeaphorn N., Hansrivijit P., Salim S.A., Bathini T., Rivera F.H.C., Cheungpasitporn W. (2020). The Use of Donor-Derived Cell-Free DNA for Assessment of Allograft Rejection and Injury Status. J. Clin. Med..

[10] Tait B.D., Süsal C., Gebel H.M., Nickerson P.W., Zachary A.A., Claas F.H., Reed E.F., Bray R.A., Campbell P., Chapman J.R. (2013). Consensus Guidelines on the Testing and Clinical Management Issues Associated with HLA and Non-HLA Antibodies in Transplantation. Transplantation.

[11] Banasik M., Boratyńska M., Kościelska-Kasprzak K., Kamińska D., Bartoszek D., Żabińska M., Myszka M., Zmonarski S., Protasiewicz M., Nowakowska B. (2014). The influence of non-HLA antibodies directed against angiotensin II type 1 receptor (AT1R) on early renal transplant outcomes. Transpl. Int..

[12] Banasik M., Jabłecki J., Boratyńska M., Kamińska D., Kościelska-Kasprzak K., Bartoszek D., Chełmoński A., Hałoń A., Baran W., Klinger M. (2014). Humoral immunity in hand transplantation: Anti-HLA and non-HLA response. Hum. Immunol..

[13] Stanimirova I., Banasik M., Ząbek A., Dawiskiba T., Kościelska-Kasprzak K., Wojtowicz W., Krajewska M., Janczak D., Mlynarz P. (2020). Serum metabolomics approach to monitor the changes in metabolite profiles following renal transplantation. Sci. Rep..

[14] Heitzer E., Auinger L., Speicher M.R. (2020). Cell-Free DNA and Apoptosis: How Dead Cells Inform About the Living. Trends Mol. Med..

[15] Mandel P., Metais P. (1948). Nuclear Acids in Human Blood Plasma. C. R. Seances Soc. Biol. Fil..

[16] Tan E.M., Schur P.H., Carr R.I., Kunkel H.G. (1966). Deoxybonucleic acid (DNA) and antibodies to DNA in the serum of patients with systemic lupus erythematosus. J. Clin. Investig..

[17] Koffler D., Agnello V., Winchester R., Kunkel H.G. (1973). The Occurrence of Single-Stranded DNA in the Serum of Patients with Systemic Lupus Erythematosus and Other Diseases. J. Clin. Investig..

[18] Leon S.A., Shapiro B., Sklaroff D.M., Yaros M.J. (1977). Free DNA in the serum of cancer patients and the effect of therapy. Cancer Res..

[19] Arneth B. (2018). Update on the types and usage of liquid biopsies in the clinical setting: A systematic review. BMC Cancer.

[20] Mouliere F., Robert B., Peyrotte E.A., Del Rio M., Ychou M., Molina F., Gongora C., Thierry A.R. (2011). High Fragmentation Characterizes Tumour-Derived Circulating DNA. PLoS ONE.

[21] Mouliere F., Chandrananda D., Piskorz A.M., Moore E.K., Morris J., Ahlborn L.B., Mair R., Goranova T., Marass F., Heider K. (2018). Enhanced detection of circulating tumor DNA by fragment size analysis. Sci. Transl. Med..

[22] Gall T.M., Belete S., Khanderia E., Frampton A.E., Jiao L.R. (2019). Circulating Tumor Cells and Cell-Free DNA in Pancreatic Ductal Adenocarcinoma. Am. J. Pathol..

[23] Kustanovich A., Schwartz R., Peretz T., Grinshpun A. (2019). Life and death of circulating cell-free DNA. Cancer Biol. Ther..

[24] Shaw J., Stebbing J. (2014). Circulating free DNA in the management of breast cancer. Ann. Transl. Med..

[25] Yu M. (2012). Circulating cell-free mitochondrial DNA as a novel cancer biomarker: Opportunities and challenges. Mitochondrial DNA.

[26] Mair R., Mouliere F., Smith C.G., Chandrananda D., Gale D., Marass F., Tsui D.W.Y., Massie C.E., Wright A.J., Watts C. (2019). Measurement of Plasma Cell-Free Mitochondrial Tumor DNA Improves Detection of Glioblastoma in Patient-Derived Orthotopic Xenograft Models. Cancer Res..

[27] Gambardella S., Limanaqi F., Ferese R., Biagioni F., Campopiano R., Centonze D., Fornai F. (2019). ccf-mtDNA as a Potential Link Between the Brain and Immune System in Neuro-Immunological Disorders. Front. Immunol..

[28] Lowes H., Pyle A., Duddy M., Hudson G. (2019). Cell-free mitochondrial DNA in progressive multiple sclerosis. Mitochondrion.

[29] Berezin A.E. (2015). Circulating Cell-Free Mitochondrial DNA as Biomarker of Cardiovascular risk: New Challenges of Old Findings. Angiol. Open Access.

[30] Akca H., Demiray A., Yaren A., Bir F., Köseler A., Iwakawa R., Bagci G., Yokota J. (2013). Utility of serum DNA and pyrosequencing for the detection of EGFR mutations in non-small cell lung cancer. Cancer Genet..

[31] Schwarzenbach H., Hoon D.S.B., Pantel K. (2011). Cell-free nucleic acids as biomarkers in cancer patients. Nat. Rev. Cancer.

[32] Alberry M., Maddocks D., Jones M., Hadi M.A., Abdel-Fattah S., Avent N., Soothill P.W. (2007). Free fetal DNA in maternal plasma in anembryonic pregnancies: Confirmation that the origin is the trophoblast. Prenat. Diagn..

[33] Chan K.C.A., Zhang J., Hui A.B.Y., Wong N., Lau T.K., Leung T.N., Lo K.-W., Huang D.W.S., Lo Y.M.D. (2004). Size Distributions of Maternal and Fetal DNA in Maternal Plasma. Clin. Chem..

[34] Rafi I., Chitty L.S. (2009). Cell-free fetal DNA and non-invasive prenatal diagnosis. Br. J. Gen. Pract..

[35] Beck J., Oellerich M., Schulz U., Schauerte V., Reinhard L., Fuchs U., Knabbe C., Zittermann A., Olbricht C., Gummert J. (2015). Donor-Derived Cell-Free DNA Is a Novel Universal Biomarker for Allograft Rejection in Solid Organ Transplantation. Transplant. Proc..

[36] Filippone E.J., Farber J.L. (2020). The Monitoring of Donor-Derived Cell-Free DNA (ddcfDNA) in Kidney Transplantation. Transplantation.

[37] Bronkhorst A.J., Ungerer V., Holdenrieder S. (2019). The emerging role of cell-free DNA as a molecular marker for cancer management. Biomol. Detect. Quantif..

[38] Diehl F., Schmidt K., Choti M.A., Romans K.E., Goodman S.N., Li M., Thornton K., Agrawal N., Sokoll L.J., Szabo S.A. (2008). Circulating mutant DNA to assess tumor dynamics. Nat. Med..

[39] Diehl F., Li M., Dressman D., He Y., Shen D., Szabo S., Diaz L.A., Goodman S.N., David K.A., Juhl H. (2005). Detection and quantification of mutations in the plasma of patients with colorectal tumors. Proc. Natl. Acad. Sci. USA.

[40] Alekseeva L.A., Mironova N.L., Brenner E.V., Kurilshikov A.M., Patutina O.A., Zenkova M.A. (2017). Alteration of the exDNA profile in blood serum of LLC-bearing mice under the decrease of tumour invasion potential by bovine pancreatic DNase I treatment. PLoS ONE.

[41] Reckamp K.L., Melnikova V.O., Karlovich C., Sequist L.V., Camidge D.R., Wakelee H., Perol M., Oxnard G.R., Kosco K., Croucher P. (2016). A Highly Sensitive and Quantitative Test Platform for Detection of NSCLC EGFR Mutations in Urine and Plasma. J. Thorac. Oncol..

[42] Chelobanov B.P., Laktionov P.P., Vlasov V.V. (2006). Proteins involved in binding and cellular uptake of nucleic acids. Biochemistry (Moscow).

[43] Ou Z., Li K., Yang T., Dai Y., Chandra M., Ning J., Wang Y., Xu R., Gao T., Xie Y. (2020). Detection of bladder cancer using urinary cell-free DNA and cellular DNA. Clin. Transl. Med..

[44] Ding S., Song X., Geng X., Liu L., Ma H., Wang X., Wei L., Xie L., Song X. (2019). Saliva-derived cfDNA is applicable for EGFR mutation detection but not for quantitation analysis in non-small cell lung cancer. Thorac. Cancer.

[45] Wang Z., Zhang L., Li L., Li X., Xu Y., Wang M., Liang L., Jiao P., Li Y., He S. (2020). Sputum Cell-Free DNA. J. Mol. Diagn..

[46] Zandvakili I., Lazaridis K.N. (2019). Cell-free DNA testing: Future applications in gastroenterology and hepatology. Ther. Adv. Gastroenterol..

[47] De Maio G. (2014). Circulating and stool nucleic acid analysis for colorectal cancer diagnosis. World J. Gastroenterol..

[48] Zinkova A., Brynychova I., Svacina A., Jirkovska M., Korabecna M. (2017). Cell-free DNA from human plasma and serum differs in content of telomeric sequences and its ability to promote immune response. Sci. Rep..

[49] Sorber L., Zwaenepoel K., Jacobs J., De Winne K., Goethals S., Reclusa P., Van Casteren K., Augustus E., Lardon F., Roeyen G. (2019). Circulating Cell-Free DNA and RNA Analysis as Liquid Biopsy: Optimal Centrifugation Protocol. Cancers.

[50] Fleischhacker M., Schmidt B., Weickmann S., Fersching D.M., Leszinski G.S., Siegele B., Stötzer O.J., Nagel D., Holdenrieder S. (2011). Methods for isolation of cell-free plasma DNA strongly affect DNA yield. Clin. Chim. Acta.

[51] Markus H., Contente-Cuomo T., Farooq M., Liang W.S., Borad M.J., Sivakumar S., Gollins S., Tran N.L., Dhruv H.D., Berens M.E. (2018). Evaluation of pre-analytical factors affecting plasma DNA analysis. Sci. Rep..

[52] Meddeb R., Pisareva E., Thierry A.R. (2019). Guidelines for the Preanalytical Conditions for Analyzing Circulating Cell-Free DNA. Clin. Chem..

[53] Lo Y.D., Tein M.S., Lau T.K., Haines C.J., Leung T.N., Poon P.M., Wainscoat J.S., Johnson P.J., Chang A.M., Hjelm N.M. (1998). Quantitative Analysis of Fetal DNA in Maternal Plasma and Serum: Implications for Noninvasive Prenatal Diagnosis. Am. J. Hum. Genet..

[54] Luger K. (2003). Structure and dynamic behavior of nucleosomes. Curr. Opin. Genet. Dev..

[55] Thierry A.R., Mouliere F., El Messaoudi S., Mollevi C., Lopez-Crapez E., Rolet F., Gillet B., Gongora C., Dechelotte P., Robert B. (2014). Clinical validation of the detection of KRAS and BRAF mutations from circulating tumor DNA. Nat. Med..

[56] Kidess E., Heirich K., Wiggin M., Vysotskaia V., Visser B.C., Marziali A., Wiedenmann B., Norton J.A., Lee M., Jeffrey S.S. (2014). Mutation profiling of tumor DNA from plasma and tumor tissue of colorectal cancer patients with a novel, high-sensitivity multiplexed mutation detection platform. Oncotarget.

[57] Birkenkamp-Demtröder K., Nordentoft I., Christensen E., Høyer S., Reinert T., Vang S., Borre M., Agerbæk M., Jensen J.B., Ørntoft T.F. (2016). Genomic Alterations in Liquid Biopsies from Patients with Bladder Cancer. Eur. Urol..

[58] Keller L., Belloum Y., Wikman H., Pantel K. (2020). Clinical relevance of blood-based ctDNA analysis: Mutation detection and beyond. Br. J. Cancer.

[59] Heitzer E., Ulz P., Belic J., Gutschi S., Quehenberger F., Fischereder K., Benezeder T., Auer M., Pischler C., Mannweiler S. (2013). Tumor-associated copy number changes in the circulation of patients with prostate cancer identified through whole-genome sequencing. Genome Med..

[60] Kinde I., Papadopoulos N., Kinzler K.W., Vogelstein B. (2012). FAST-SeqS: A Simple and Efficient Method for the Detection of Aneuploidy by Massively Parallel Sequencing. PLoS ONE.

[61] Newman A.M., Lovejoy A.F., Klass D.M., Kurtz D.M., Chabon J.J., Scherer F., Stehr H., Liu C.L., Bratman S.V., Say C. (2016). Integrated digital error suppression for improved detection of circulating tumor DNA. Nat. Biotechnol..

[62] Gale D., Lawson A.R.J., Howarth K., Madi M., Durham B., Smalley S., Calaway J., Blais S., Jones G., Clark J. (2018). Development of a highly sensitive liquid biopsy platform to detect clinically-relevant cancer mutations at low allele fractions in cell-free DNA. PLoS ONE.

[63] Kinde I., Wu J., Papadopoulos N., Kinzler K.W., Vogelstein B. (2011). Detection and quantification of rare mutations with massively parallel sequencing. Proc. Natl. Acad. Sci. USA.

[64] Verhoeven J.G.H.P., Boer K., Van Schaik R.H.N., Belardi J.A., Huibers M.M.H., Baan C.C., Hesselink D.A. (2018). Liquid Biopsies to Monitor Solid Organ Transplant Function. Ther. Drug Monit..

[65] Lo Y.D., Tein M.S., Pang C.C., Yeung C.K., Tong K.-L., Hjelm N.M. (1998). Presence of donor-specific DNA in plasma of kidney and liver-transplant recipients. Lancet.

[66] Gadi V.K., Nelson J.L., Boespflug N.D., Guthrie K.A., Kuhr C.S. (2006). Soluble Donor DNA Concentrations in Recipient Serum Correlate with Pancreas-Kidney Rejection. Clin. Chem..

[67] Oellerich M., Walson P.D., Beck J., Schmitz J., Kollmar O., Schütz E. (2016). Graft-Derived Cell-Free DNA as a Marker of Transplant Graft Injury. Ther. Drug Monit..

[68] Sharon E., Shi H., Kharbanda S., Koh W., Martin L.R., Khush K.K., Valantine H., Pritchard J.K., De Vlaminck I. (2017). Quantification of transplant-derived circulating cell-free DNA in absence of a donor genotype. PLoS Comput. Biol..

[69] Gordon P., Khan A., Sajid U., Chang N., Suresh V., Dimnik L., Lamont R.E., Parboosingh J.S., Martin S.R., Pon R.T. (2016). An Algorithm Measuring Donor Cell-Free DNA in Plasma of Cellular and Solid Organ Transplant Recipients That Does Not Require Donor or Recipient Genotyping. Front. Cardiovasc. Med..

[70] Grskovic M., Hiller D.J., Eubank L.A., Sninsky J.J., Christopherson C., Collins J.P., Thompson K., Song M., Wang Y.S., Ross D. (2016). Validation of a Clinical-Grade Assay to Measure Donor-Derived Cell-Free DNA in Solid Organ Transplant Recipients. J. Mol. Diagn..

[71] Dengu F., Mason P. (2020). Next-generation sequencing methods to detect donor-derived cell-free DNA after transplantation. Transplant. Rev..

[72] Snyder T.M., Khush K.K., Valantine H.A., Quake S.R. (2011). Universal noninvasive detection of solid organ transplant rejection. Proc. Natl. Acad. Sci. USA.

[73] De Vlaminck I., Valantine H.A., Snyder T.M., Strehl C., Cohen G., Luikart H., Neff N.F., Okamoto J., Bernstein D., Weisshaar D. (2014). Circulating Cell-Free DNA Enables Noninvasive Diagnosis of Heart Transplant Rejection. Sci. Transl. Med..

[74] Beck J., Bierau S., Balzer S., Andag R., Kanzow P., Schmitz J., Gaedcke J., Moerer O., Slotta J.E., Walson P. (2013). Digital Droplet PCR for Rapid Quantification of Donor DNA in the Circulation of Transplant Recipients as a Potential Universal Biomarker of Graft Injury. Clin. Chem..

[75] De Vlaminck I., Martin L., Kertesz M., Patel K., Kowarsky M., Strehl C., Cohen G., Luikart H., Neff N.F., Okamoto J. (2015). Noninvasive monitoring of infection and rejection after lung transplantation. Proc. Natl. Acad. Sci. USA.

[76] Krenzien F., Katou S., Papa A., Sinn B., Benzing C., Feldbrügge L., Kamali C., Brunnbauer P., Splith K., Lorenz R.R. (2020). Increased Cell-Free DNA Plasma Concentration Following Liver Transplantation Is Linked to Portal Hepatitis and Inferior Survival. J. Clin. Med..

[77] Schütz E., Fischer A., Beck J., Harden M., Koch M., Wuensch T., Stockmann M., Nashan B., Kollmar O., Matthaei J. (2017). Graft-derived cell-free DNA, a noninvasive early rejection and graft damage marker in liver transplantation: A prospective, observational, multicenter cohort study. PLoS Med..

[78] Sayah D.M., Weigt S.S., Ramsey A., Ardehali A., Golden J., Ross D.J. (2020). Plasma Donor-derived Cell-free DNA Levels Are Increased During Acute Cellular Rejection After Lung Transplant: Pilot Data. Transplant. Direct.

[79] Valapour M., Skeans M.A., Smith J.M., Edwards L.B., Cherikh W.S., Callahan E.R., Israni A.K., Snyder J.J., Kasiske B.L. (2016). Lung. Arab. Archaeol. Epigr..

[80] Zou J., Duffy B., Slade M., Young A.L., Steward N., Hachem R., Mohanakumar T. (2017). Rapid detection of donor cell free DNA in lung transplant recipients with rejections using donor-recipient HLA mismatch. Hum. Immunol..

[81] Richmond M.E., Zangwill S.D., Kindel S.J., Deshpande S.R., Schroder J.N., Bichell D.P., Knecht K.R., Mahle W.T., Wigger M.A., Gaglianello N.A. (2020). Donor fraction cell-free DNA and rejection in adult and pediatric heart transplantation. J. Heart Lung Transplant..

[82] Khush K.K., Patel J., Pinney S., Kao A., Alharethi R., DePasquale E., Ewald G., Berman P., Kanwar M., Hiller D. (2019). Noninvasive detection of graft injury after heart transplant using donor-derived cell-free DNA: A prospective multicenter study. Arab. Archaeol. Epigr..

[83] AlRashidi F.T., Gillespie K.M. (2018). Biomarkers in Islet Cell Transplantation for Type 1 Diabetes. Curr. Diabetes Rep..

[84] Gala-Lopez B., Neiman D., Kin T., O’Gorman D., Pepper A.R., Malcolm A.J., Pianzin S., Senior P.A., Campbell P., Glaser B. (2018). Beta Cell Death by Cell-free DNA and Outcome After Clinical Islet Transplantation. Transplantation.

[85] Brunet M., Van Gelder T., Åsberg A., Haufroid V., Hesselink D.A., Langman L., Lemaitre F., Marquet P., Seger C., Shipkova M. (2019). Therapeutic Drug Monitoring of Tacrolimus-Personalized Therapy. Ther. Drug Monit..

[86] Klinger M., Banasik M. (2015). Immunological characteristics of the elderly allograft recipient. Transplant. Rev..

[87] Banasik M., Klinger M. (2006). Chronic allograft nephropathy--immunologic and nonimmunologic factors. Ann. Transplant..

[88] Bloom R.D., Bromberg J.S., Poggio E.D., Bunnapradist S., Langone A.J., Sood P., Matas A.J., Mehta S., Mannon R.B., Sharfuddin A. (2017). Cell-Free DNA and Active Rejection in Kidney Allografts. J. Am. Soc. Nephrol..

[89] Huang E., Sethi S., Peng A., Najjar R., Mirocha J., Haas M., Vo A., Jordan S.C. (2019). Early clinical experience using donor-derived cell-free DNA to detect rejection in kidney transplant recipients. Arab. Archaeol. Epigr..

[90] Stites E., Kumar D., Olaitan O., Swanson S.J., Leca N., Weir M., Bromberg J.S., Melancon J., Agha I., Fattah H. (2020). High levels of dd-cfDNA identify patients with TCMR 1A and borderline allograft rejection at elevated risk of graft injury. Arab. Archaeol. Epigr..

[91] Gielis E.M., Ledeganck K.J., Dendooven A., Meysman P., Beirnaert C., Laukens K., De Schrijver J., Van Laecke S., Van Biesen W., Emonds M.-P. (2019). The use of plasma donor-derived, cell-free DNA to monitor acute rejection after kidney transplantation. Nephrol. Dial. Transplant..

[92] Gielis E.M., Beirnaert C., Dendooven A., Meysman P., Laukens K., De Schrijver J., Van Laecke S., Van Biesen W., Emonds M.-P., De Winter B.Y. (2018). Plasma donor-derived cell-free DNA kinetics after kidney transplantation using a single tube multiplex PCR assay. PLoS ONE.

[93] Sigdel T., Archila F.A., Constantin T., Demko Z.P., Liberto J.M., Damm I., Towfighi P., Navarro S., Kirkizlar E., Demko Z. (2018). Optimizing Detection of Kidney Transplant Injury by Assessment of Donor-Derived Cell-Free DNA via Massively Multiplex PCR. J. Clin. Med..

[94] Zhang H., Zheng C., Li X., Fu Q., Li J., Su Q., Zeng L., Liu Z., Wang J., Huang H. (2020). Diagnostic Performance of Donor-Derived Plasma Cell-Free DNA Fraction for Antibody-Mediated Rejection in Post Renal Transplant Recipients: A Prospective Observational Study. Front. Immunol..

[95] Hurkmans D.P., Verhoeven J.G.H.P., De Leur K., Boer K., Joosse A., Baan C.C., Von Der Thüsen J.H., Van Schaik R., Mathijssen R.H.J., Van Der Veldt A.A. (2019). Donor-derived cell-free DNA detects kidney transplant rejection during nivolumab treatment. J. Immunother. Cancer.

[96] Whitlam J.B., Ling L., Skene A., Kanellis J., Ierino F.L., Slater H., Bruno D.L., Power D.A. (2019). Diagnostic application of kidney allograft-derived absolute cell-freeDNAlevels during transplant dysfunction. Arab. Archaeol. Epigr..

[97] Shen J., Guo L., Yan P., Zhou J., Zhou Q., Lei W., Liu H., Liu G., Lv J., Liu F. (2020). Prognostic value of the donor-derived cell-free DNA assay in acute renal rejection therapy: A prospective cohort study. Clin. Transplant..

[98] Zhou Y., Yang G., Liu H., Chen Y., Li X., Ge J., Wang X., Niu H., Dong W., Jiang T. (2019). A Noninvasive and Donor-independent Method Simultaneously Monitors Rejection and Infection in Patients with Organ Transplant. Transplant. Proc..

[99] Watson D., Yang J.S., Sarwal R.D., Sigdel T., Liberto J.M., Damm I., Louie V., Sigdel S., Livingstone D., Soh K. (2019). A Novel Multi-Biomarker Assay for Non-Invasive Quantitative Monitoring of Kidney Injury. J. Clin. Med..

[100] Schena F.P., Nistor I. (2018). Epidemiology of IgA Nephropathy: A Global Perspective. Semin. Nephrol..

[101] Yang J.Y., Sarwal R.D., Fervenza F.C., Sarwal M.M., Lafayette R.A. (2019). Noninvasive Urinary Monitoring of Progression in IgA Nephropathy. Int. J. Mol. Sci..

[102] Yang J.Y., Sarwal R.D., Sigdel T.K., Damm I., Rosenbaum B., Liberto J.M., Chan-On C., Arreola-Guerra J.M., Alberu J., Vincenti F. (2020). A urine score for noninvasive accurate diagnosis and prediction of kidney transplant rejection. Sci. Transl. Med..

[103] Nolan N., Valdivieso K., Mani R., Yang J.Y., Sarwal R.D., Katzenbach P., Chalasani K., Hongo D., Lugtu G., Mark C. (2020). Clinical and Analytical Validation of a Novel Urine-Based Test for the Detection of Allograft Rejection in Renal Transplant Patients. J. Clin. Med..

[104] Abbott K.C., Swanson S., Richter E.R., Bohen E.M., Agodoa L.Y., Peters T.G., Barbour G., Lipnick R., Cruess D.F. (2004). Late urinary tract infection after renal transplantation in the United States. Am. J. Kidney Dis..

[105] Burnham P., Dadhania D., Heyang M., Chen F., Westblade L.F., Suthanthiran M., Lee J.R., De Vlaminck I. (2018). Urinary cell-free DNA is a versatile analyte for monitoring infections of the urinary tract. Nat. Commun..

[106] Sigdel T.K., Vitalone M.J., Tran T.Q., Dai H., Hsieh S.-C., Salvatierra O., Sarwal M.M. (2013). A Rapid Noninvasive Assay for the Detection of Renal Transplant Injury. Transplantation.

[107] Homolová J., Janovičová Ľ., Konečná B., Vlkova B., Celec P., Tothova L., Bábíčková J. (2020). Plasma Concentrations of Extracellular DNA in Acute Kidney Injury. Diagnostics.

[108] El Tarhouny S.A., Hadhoud K.M., Ebrahem M.M., Al Azizi N.M. (2010). Assessment of Cell-Free DNA with Microvascular Complication of Type II Diabetes Mellitus, Using PCR and Elisa. Nucleosides Nucleotides Nucleic Acids.

[109] Li X., Hu R., Luo T., Peng C., Gong L., Hu J., Yang S., Li Q. (2020). Serum cell-free DNA and progression of diabetic kidney disease: A prospective study. BMJ Open Diabetes Res. Care.

[110] Chang C.-C., Chiu P.-F., Wu C.-L., Kuo C.-L., Huang C.-S., Liu C.-S. (2019). Urinary cell-free mitochondrial and nuclear deoxyribonucleic acid correlates with the prognosis of chronic kidney diseases. BMC Nephrol..

[111] Ponti G., Manfredini M., Tomasi A. (2019). Non-blood sources of cell-free DNA for cancer molecular profiling in clinical pathology and oncology. Crit. Rev. Oncol..

[112] Lasseter K., Nassar A.H., Hamieh L., Berchuck J.E., Nuzzo P.V., Korthauer K., Shinagare A.B., Ogorek B., McKay R., Thorner A.R. (2020). Plasma cell-free DNA variant analysis compared with methylated DNA analysis in renal cell carcinoma. Genet. Med..

[113] Bacon J.V., Annala M., Soleimani M., Lavoie J.-M., So A., Gleave M.E., Fazli L., Wang G., Chi K.N., Kollmannsberger C.K. (2020). Plasma Circulating Tumor DNA and Clonal Hematopoiesis in Metastatic Renal Cell Carcinoma. Clin. Genitourin. Cancer.

[114] Rouvinov K., Mermershtain W., Dresler H., Ariad S., Riff R., Shani-Shrem N., Keizman D., Douvdevani A. (2017). Circulating Cell-Free DNA Levels in Patients with Metastatic Renal Cell Carcinoma. Oncol. Res. Treat..

[115] Skrypkina I., Tsyba L., Onyshchenko K., Morderer D., Kashparova O., Nikolaienko O., Panasenko G., Vozianov S., Romanenko A., Rynditch A. (2016). Concentration and Methylation of Cell-Free DNA from Blood Plasma as Diagnostic Markers of Renal Cancer. Dis. Markers.

[116] Lu H., Busch J., Jung M., Rabenhorst S., Ralla B., Kilic E., Mergemeier S., Budach N., Fendler A., Jung K. (2016). Diagnostic and prognostic potential of circulating cell-free genomic and mitochondrial DNA fragments in clear cell renal cell carcinoma patients. Clin. Chim. Acta.

